# A rare presentation of primary lung adenocarcinoma mimicking bilateral interstitial infiltration: A case report and literature review

**DOI:** 10.1016/j.ijscr.2025.110899

**Published:** 2025-01-15

**Authors:** Renad Abed, Wasef Alhroub, Yousef Abu Asbeh, Abdelrahman Rabee, Sami Bannoura, Arein Madia

**Affiliations:** a Faculty of Medicine, Hebron University, Hebron, Palestine; b Al-Ahli hospital, Hebron, Palestine; c College of Medicine, Hebron University, Hebron 40, Palestine; d Department of pathology, Al-Ahli hospital, Hebron, Palestine; e College of Medicine, Hebron, Palestine

**Keywords:** Lung adenocarcinoma, Interstitial Lund disease, Micopapillary, Interstitial infiltration, Mimicking

## Abstract

**Background:**

Primary lung adenocarcinoma can sometimes present atypically, mimicking interstitial lung disease (ILD), and posing significant diagnostic challenges. Such presentations often lead to misdiagnoses, delaying appropriate treatment.

**Case presentation:**

A 35-year-old female non-smoker presented with a six-month history of progressive cough, mild hemoptysis, fatigue, and exertional dyspnea, with no associated weight loss. Imaging studies revealed diffuse ground-glass opacities and interstitial infiltrates, while pulmonary function tests were consistent with interstitial lung disease. Despite these findings, bronchoscopy results were normal. A definitive diagnosis was ultimately made through a biopsy, which identified a moderately to poorly differentiated adenocarcinoma with acinar and micropapillary features.

**Discussion:**

This case highlights the diagnostic complexity when lung adenocarcinoma presents atypically, mimicking ILD. Conventional diagnostic tools, such as imaging and pulmonary function tests, may overlap with ILD findings, leading to misdiagnoses. Early consideration of malignancy and the use of invasive diagnostic procedures, such as biopsy, are essential for distinguishing between ILD and malignancy in atypical cases.

**Conclusion:**

This case underscores the importance of maintaining a high index of suspicion for malignancy in atypical ILD presentations. Early invasive diagnostic techniques are crucial for achieving a timely and accurate diagnosis, ultimately improving patient outcomes.

## Highlights

• Primary lung adenocarcinoma can present in ways that mimic interstitial lung disease (ILD), complicating diagnosis and potentially delaying appropriate treatment.
• Atypical presentations of lung adenocarcinoma can lead to misdiagnosis, as imaging and pulmonary function tests may resemble those of ILD.
• Despite normal bronchoscopy findings, further invasive diagnostic procedures, such as biopsy, are essential for establishing an accurate diagnosis in cases of suspected ILD.
• Lung adenocarcinoma may present with ground-glass opacities and interstitial infiltrates, which are commonly associated with ILD, emphasizing the need for careful evaluation.
• Maintaining a high index of suspicion for malignancy in cases of atypical ILD is critical, as early intervention with invasive diagnostics can improve patient outcomes.

## 1 Introduction

This work has been reported in line with the SCARE criteria [1].

Lung cancer is the most common cause of cancer-related deaths worldwide. There are two types of lung cancer: small cell lung cancer (SCLC) and non-small cell lung cancer (NSCLC) which account for around 85 % of all lung cancers [2].

Adenocarcinomas may present on imaging as bilateral nodular, interstitial, or ground glass opacities, or less commonly, as unilateral solid or ground glass nodules, or masses. When there is no history of smoking, these opacities could be initially misinterpreted as inflammatory or infectious or other any pulmonary diseases like interstitial lung disease, which frequently delays the diagnosis [3]. Interstitial lung diseases (ILDs) encompass a diverse group of diffuse parenchymal lung disorders with overlapping radiologic and clinical features, making differentiation from malignancy challenging. Accurate and prompt diagnosis is crucial, as delays in identifying malignancy, such as lung adenocarcinoma, can adversely affect patient outcomes. A tissue biopsy is the definitive diagnostic tool, providing critical histopathological evidence to distinguish between benign and malignant conditions, ensuring appropriate management [4].

We present a challenging diagnostic case of a 35-year-old female non-smoker who exhibited diffuse pulmonary infiltrates on imaging. Despite initial suspicion of ILD, further investigations revealed a diagnosis of primary lung adenocarcinoma with acinar and micropapillary features. This case underscores the diagnostic complexities associated with primary lung adenocarcinoma, which can manifest as bilateral interstitial infiltrates, often resembling various pulmonary diseases. While typical lung cancer presentations are generally straightforward to identify, atypical presentations can significantly complicate the diagnostic process.

## 2 Case presentation

**Fig. 1:**
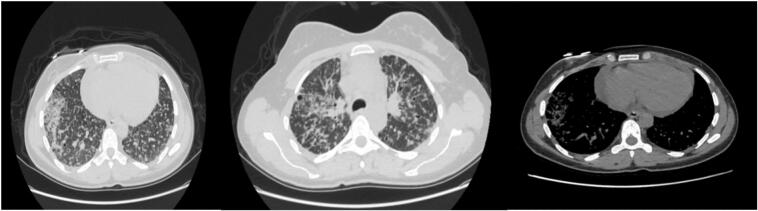
CT chest axial images showing diffuse bilateral interstitial infiltrates, intermixed with ground glass infiltrates and some cystic changes.

**Fig. 2:**
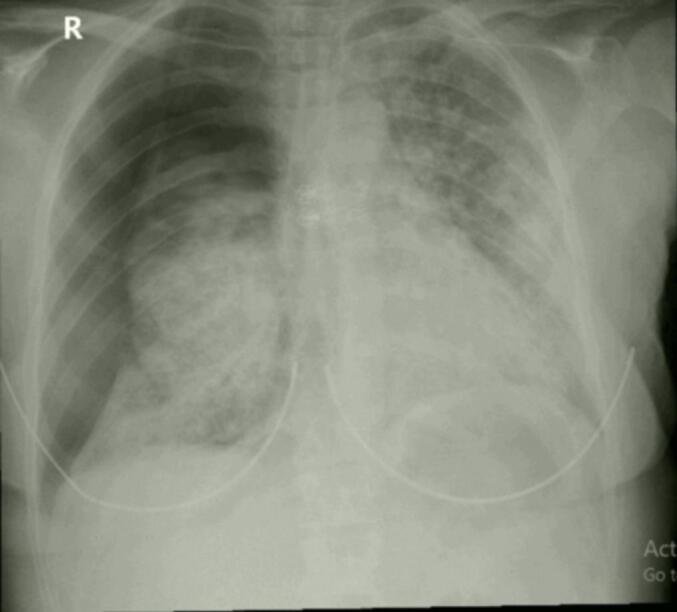
chest X-ray (CXR) showing a right pneumothorax, prominent diffuse bilateral interstitial alveolar opacities.

**Fig. 3:**
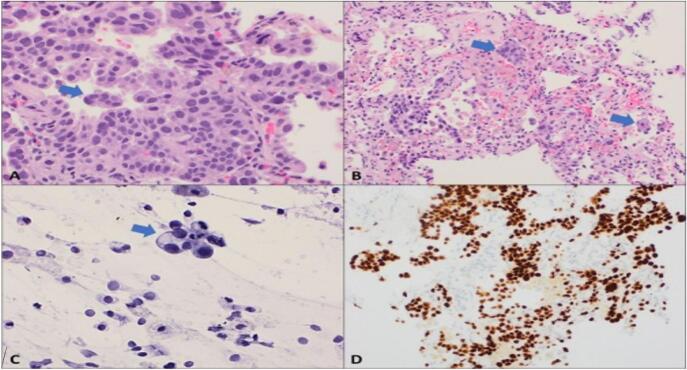
Moderately differentiated pulmonary adenocarcinoma with micropapillary component; A. Section shows pulmonary adenocarcinoma with acinar and micropapillary morphology (arrow) (H&E, 40×); B. Tumor cell clusters within alveolar spaces (arrow) (H&E, 20×); C. Bronchoalveolar lavage positive for malignant cell clusters (arrow) (40×); D. The tumor cells are positive for TTF1 immunostaining (20×).

A 35-year-old Palestinian female non-smoker, previously healthy, presented with a six-month history of progressive cough, mild hemoptysis, increasing fatigue, and dyspnea with mild-to-moderate exertion, without any associated weight loss. On admission, her vital signs were stable, and her BMI was 23.4. She is a housewife with no history of chronic illnesses, surgeries, or drug use and no occupational exposure to toxins. Her family history was unremarkable. Physical examination showed clear lungs with good bilateral air entry on auscultation and no abnormalities in the cardiovascular, abdominal, or neurological systems. There was no lymphadenopathy, clubbing, cyanosis, or peripheral edema, and the overall examination was normal. The patient sought treatment for her cough over several months from various specialists. Afterward, she was referred to a pulmonologist, whose high-resolution chest CT scan indicated bilateral reticulonodular infiltrates with cystic changes suggestive of interstitial lung disease (ILD) (Fig. 1). Pulmonary function tests showed forced vital capacity (FVC) of 1.63 l, forced expiratory volume in 1 s (FEV1) of 1.64 l, and an FVC/FEV1 ratio of 1.01. Based on these findings, the patient was admitted for bronchoscopy to further evaluate the possibility of ILD, but the procedure revealed normal right and left bronchi with no endobronchial lesions or active hemoptysis. Laboratory tests, including complete blood count and comprehensive metabolic panel, were normal except for an elevated CRP of 8.9. Bronchoalveolar lavage and multiple transbronchial biopsies from the right upper and middle lobes were performed for histopathological analysis. The patient later developed an iatrogenic right-sided pneumothorax, which was confirmed on chest X-ray (Fig. 2). A right posterolateral chest tube was placed, and the pneumothorax resolved the following day. Cytology and pathology revealed fragments of moderately-to-poorly differentiated pulmonary adenocarcinoma with acinar and micropapillary features. Tumor cells were positive for TTF1 immunostaining (Fig. 3).

Following that, a positron emission tomography (PET) scan revealed hypermetabolic malignant bilateral pulmonary areas with dense consolidations, nodular ground-glass opacities, and lymphangitic spread, along with metastatic left hilar lymph nodes, confirming the primary tumor. The patient underwent video-assisted thoracoscopic surgery (VATS), which included a wedge resection from the right middle lobe and a biopsy from the right upper lobe, further confirming moderately-to-poorly differentiated pulmonary adenocarcinoma. A pericardial window was also created. Genetic testing identified a pathogenic ERBB (HER2) mutation, while KRAS and ALK were negative.

The patient completed 10 cycles of chemotherapy with carboplatin and pemetrexed. After six months, she reported no improvement in her symptoms, including persistent hemoptysis. A comparison of CT scans revealed disease progression, with an increase in the size and number of bilateral pulmonary nodules, characterized by ground-glass opacities and diffuse septal thickening. There were also multiple bilateral pulmonary cysts up to 13 mm, apical fibrotic changes, and mild right-sided pleural effusion.

## 3 Discussion

Lung adenocarcinoma represents about 40 % of all lung cancers and is associated with a high mortality rate. It commonly spreads to the liver, adrenal glands, brain, and bones, but rarely to soft tissues [5,6]. It is the most common primary lung cancer in the United States and is a subtype of non-small cell lung cancer (NSCLC). While strongly linked to smoking, it is also the most frequent lung cancer in individuals who have never smoked. Typically originating in the lung periphery, it often arises from mucosal glands and may develop in areas of scarring or chronic inflammation. Despite declining incidence and mortality rates, it remains the leading cause of cancer-related deaths in the U.S [7]. It presents with diverse clinical features, ranging from small solitary or multiple nodules to extensive miliary patterns or diffuse infiltrates resembling bacterial pneumonia. Its pathogenesis remains under investigation, but tumor proliferation is often accompanied by significant inflammation and fibrosis that mimic benign inflammatory conditions. This resemblance can delay diagnosis and negatively impact patients' quality of life [8]. Retrospective studies show that younger lung cancer patients are more likely to present with adenocarcinoma, be female, and have advanced-stage disease [9,10].

The ERBB2 (HER2) mutation in lung adenocarcinoma is a relatively uncommon alteration that is associated with aggressive tumor behavior, poor prognosis, and resistance to standard chemotherapy. This mutation results in the continuous activation of HER2 signaling pathways, which drive cell proliferation and survival. In cases like ours, the presence of this mutation can complicate diagnosis and delay treatment. However, targeted therapies such as trastuzumab-deruxtecan (T-DXd), neratinib, and trastuzumab have shown promising results in HER2-mutant lung cancer, leading to improved patient outcomes when compared to conventional therapies [11].

Lung cancer is the leading cause of cancer-related deaths globally. Before the 2015 update to the World Health Organization (WHO) classification, lung adenocarcinoma was diagnosed based on acinar or tubular patterns, mucin production, and the expression of TTF-1 and Napsin A [6,12]. In 2015, the WHO adopted a new classification of lung adenocarcinoma based on histological subtypes [12]. Lung adenocarcinoma is classified based on the predominant histological pattern, with subtypes quantified in 5 % increments due to variability in both subtype and proportion. Prognosis varies significantly by subtype, even at the same stage. The lepidic predominant subtype, considered low-grade, has a favorable prognosis, while acinar and papillary subtypes are intermediate grade [13]. Acinar patterns prognosis are considered better than micropapillary, mucinous/colloid, and solid patterns, but worse than lepidic predominant patterns [14,15]. In contrast, micropapillary and solid predominant subtypes, which are high-grade patterns, are associated with a worse prognosis, even after curative resection in early-stage lung adenocarcinoma [13]. High-grade patterns are linked to smoking, lymphatic vessel invasion (LVI), and nonground-glass opacity (GGO) lesions, all of which are important prognostic factors in lung cancer [16].

Lung cancer can mimic various benign conditions, such as pneumonia, lung abscess, postinfectious scarring, atelectasis, mediastinal masses, emphysema, and granulomatous diseases. On computed tomography (CT), lung adenocarcinoma may present as ground-glass nodules, consolidative opacities, or solid mass lesions [17]. Lung cancer and ILD involve distinct biological pathways, though the precise genetic and cellular mechanisms linking these conditions remain poorly understood [18].

The patient's initial presentation, characterized by bilateral interstitial and ground-glass opacities on imaging, pulmonary function tests indicative of an ILD pattern, and normal bronchoscopy findings, led to an initial misdiagnosis and treatment for ILD. The overlapping clinical and radiological features highlight the difficulty of differentiating malignancy from benign conditions, especially in the absence of overt risk factors or symptoms specific to lung cancer.

The differential diagnosis of our case includes interstitial lung diseases (e.g., usual interstitial pneumonia, cryptogenic organizing pneumonia), infectious processes (e.g., atypical pneumonia, fungal infections), and malignancies that mimic ILD, such as lymphangitic carcinomatosis. Non-malignant mimics, such as drug-induced or hypersensitivity pneumonitis, should also be considered. Imaging, which can reveal patterns like ground-glass opacities or reticular infiltrates, and histological confirmation via biopsy are important diagnostic tools for differentiating between inflammatory, infectious, and neoplastic diseases.

Cengiz et al. describe a 74-year-old male non-smoker presenting with progressive dyspnea and persistent cough. Imaging revealed bilateral interstitial changes in the lungs, initially suggestive of interstitial lung disease (ILD). However, a transthoracic lung biopsy showed histological features, including lepidic and micropapillary patterns, leading to a diagnosis of minimally invasive adenocarcinoma of the lung [19]. Similarly, Mehta et al. report a 59-year-old woman with a history of smoking and environmental exposures who presented with worsening respiratory symptoms, including dyspnea, cough, and weight loss. Initial findings pointed to ILD, and steroid therapy was initiated with limited improvement. A subsequent biopsy confirmed bronchoalveolar carcinoma characterized by a lepidic growth pattern and cuboidal cells lining the alveoli, establishing the diagnosis of primary lung adenocarcinoma [4].

Further studies have provided valuable insights into micropapillary carcinoma. Hung et al. analyzed the prognostic significance of clinicopathological variables for metastasis-free survival in lung adenocarcinoma with distant metastases. Among 182 patients, common metastatic sites included contralateral lung (51.1 %), brain (44.5 %), bone (39.0 %), and liver (8.9 %). The micropapillary variant was strongly linked to brain metastasis (hazard ratio (HR), 2.686) and reduced brain MFS (HR, 2.186). This highlights the distinct metastatic patterns of lung adenocarcinoma subtypes and underscores the need for subtype-specific follow-up strategies. Further research is required to confirm these findings [20]. A study by Luo et al. examined patients with stage IB invasive adenocarcinoma and found that adjuvant chemotherapy improved disease-free survival. Notably, in cases with micropapillary or solid patterns, adjuvant chemotherapy significantly enhanced DFS, although it did not lead to an improvement in overall survival. This highlights the potential benefit of chemotherapy in specific histological subtypes of lung adenocarcinoma [21].

## 4 Conclusion

This case underscores the challenges in diagnosing primary lung adenocarcinoma when it presents as bilateral interstitial infiltrates, an uncommon presentation. The overlap of symptoms with interstitial lung disease necessitates a high index of suspicion, especially in non-smokers with unresponsive pulmonary symptoms. Clinicians should maintain a high level of suspicion and consider malignancy when presented with atypical pulmonary infiltrates, even in non-smokers. Prompt histopathological evaluation, including biopsy, is crucial for distinguishing malignancy from other conditions. Timely and precise diagnosis ensures proper treatment planning, reduces unnecessary interventions, and ultimately improves patient outcomes. Managing such cases requires a multidisciplinary approach, bringing together pulmonologists, oncologists, radiologists, and pathologists to ensure thorough evaluation and personalized treatment strategies. Increased familiarity with these rare manifestations of lung cancer will enhance diagnostic accuracy and optimize management, helping clinicians better navigate complex cases and provide more effective care.

## Informed consent

The patient provided written informed consent for the publication of this case report and associated images. A copy of the consent form is available for review by the Editor-in-Chief of this journal upon request.

## Ethics approval

This research did not require ethical approval, as the IRB committee does not mandate approval for reporting individual cases or case series.

## Funding

This research was not supported by any specific grants from public, commercial, or not-for-profit funding agencies.

## Declaration of competing interest

None.

## Data Availability

All data supporting the findings of this study are included in the article and are easily accessible.

## References

[1] Sohrabi C., Mathew G., Maria N., Kerwan A., Franchi T., Agha R.A. (2023). The SCARE 2023 guideline: updating consensus surgical CAse REport (SCARE) guidelines. Int. J. Surg..

[2] Kocher F. (2015). Longitudinal analysis of 2293 NSCLC patients: a comprehensive study from the TYROL registry. Lung Cancer.

[3] M. Ismail and R. Sekhon, “Diffuse pulmonary infiltrates: A guise of adenocarcinoma.,” *Respiratory medicine case reports*, vol. 22. England, pp. 150–153, 2017. doi: 10.1016/j.rmcr.2017.08.003. PMC5552382 28831374

[4] Mehta A., Bath A., Gadre A., Schauer M. (2020). Lung adenocarcinoma presenting as interstitial lung disease. BMJ Case Rep..

[5] Wang J. (2022). Case report: lung adenocarcinoma initially presenting with cutaneous and subcutaneous metastases. Front. Oncol..

[6] Jabareen M., Aljaradat A., Natsheh M., Asbeh Y.A., Shawar H. (2024). Collision tumor of pulmonary adenocarcinoma and small lymphocytic lymphoma: a rare case of concurrent malignancies in the same lymph node. Int. J. Surg. Case Rep..

[7] Li C., Lu H. (2018). Adenosquamous carcinoma of the lung. Onco. Targets. Ther..

[8] Lantuejoul S., Colby T.V., Ferretti G.R., Brichon P.Y., Brambilla C., Brambilla E. (2004). Adenocarcinoma of the lung mimicking inflammatory lung disease with honeycombing. Eur. Respir. J..

[9] Subramanian J. (2010). Distinctive characteristics of non-small cell lung cancer (NSCLC) in the young: a surveillance, epidemiology, and end results (SEER) analysis. J. Thorac. Oncol..

[10] Chen K.Y., Chang C.H., Yu C.J., Kuo S.H., Yang P.C. (2005). Distribution according to histologic type and outcome by gender and age group in Taiwanese patients with lung carcinoma. Cancer.

[11] Uy N.F., Merkhofer C.M., Baik C.S. (2022). HER2 in non-small cell lung Cancer: a review of emerging therapies. Cancers (Basel).

[12] Inamura K. (2018). Update on immunohistochemistry for the diagnosis of lung cancer. Cancers (Basel)..

[13] Yanagawa N., Shiono S., Abiko M., Katahira M., Osakabe M., Ogata S.Y. (2016). The clinical impact of solid and micropapillary patterns in resected lung adenocarcinoma. J. Thorac. Oncol..

[14] Tang E.R., Schreiner A.M., Pua B.B. (2014). Advances in lung adenocarcinoma classification: a summary of the new international multidisciplinary classification system (IASLC/ATS/ERS). J. Thorac. Dis..

[15] Russell P.A., Wainer Z., Wright G.M., Daniels M., Conron M., Williams R.A. (2011). Does lung adenocarcinoma subtype predict patient survival?: a clinicopathologic study based on the new international association for the study of lung cancer/American thoracic society/European respiratory society international multidisciplinary lung adeno. J. Thorac. Oncol..

[16] Mäkinen J.M. (2017). Histological features of malignancy correlate with growth patterns and patient outcome in lung adenocarcinoma. Histopathology.

[17] Detterbeck F.C. (2016). The IASLC lung cancer staging project: background data and proposals for the application of TNM staging rules to lung cancer presenting as multiple nodules with ground glass or lepidic features or a pneumonic type of involvement in the forthcoming eighth. J. Thorac. Oncol..

[18] Griese M. (2023). Interstitial lung diseases. Monatsschr. Kinderheilkd..

[19] Cengiz S.K., Kıral N., Baysal T., Geçmen G.G. (2024). Adenocarcinoma of the lung mimicking interstitial lung disease. Gulhane Med. J..

[20] Hung J.J., Jeng W.J., Wu Y.C., Chou T.Y., Hsu W.H. (2016). Factors predicting organ-specific distant metastasis in patients with completely resected lung adenocarcinoma. Oncotarget.

[21] Luo J. (2016). Prognostic and predictive value of the novel classification of lung adenocarcinoma in patients with stage IB. J. Cancer Res. Clin. Oncol..

